# Response to Comment on “*ApoE* e4e4 Genotype and Mortality With COVID-19 in UK Biobank” by Kuo et al.

**DOI:** 10.1093/gerona/glaa198

**Published:** 2020-08-14

**Authors:** Chia-Ling Kuo, David Melzer

**Affiliations:** 1 Connecticut Convergence Institute for Translation in Regenerative Engineering, University of Connecticut Health, Farmington, Connecticut; 2 Center on Aging, University of Connecticut Health, Farmington, Connecticut; 3 Epidemiology and Public Health Group, University of Exeter Medical School, Exeter, Devon, UK

Nikogosov et al. questions 2 main issues related to our *APOE* and COVID-19 letters (1,2). These focus on the initial candidate gene analysis reporting an association which was not genome-wide significant, with the suggestion that this somehow undermines genome-wide association studies (GWAS). Secondly, our choice of control group is questioned.

On the first issue, our work was undertaken after the COVID-19 Host Genetics Initiative (https://www.covid19hg.org/) was set up to enable pooled GWAS analyses of COVID-19 host genetics. Given that dementia had emerged as a major risk factor for severe COVID-19 (3), we hypothesized that the dementia risk haplotype *APOE* e4 could be an important factor. Genome-wide association studies are designed to test single base variant associations, usually employing additive models. Additional analyses are therefore needed to analyze complex haplotypes like those of *APOE*, combining 2 separate variants and requiring genotype-based analyses with no presumption of the inheritance mode, which are not part of the standard GWAS analysis. We therefore undertook this additional analysis, with no risk of undermining the GWAS effort for COVID-19.

The authors point out that the *APOE* e4e4 association we reported reached genome-wide significance (*p* < 5 × 10^–8^) in our second analysis after additional data became available, but not in the first. We agree that under normal circumstances, it would not have been ideal to publish non-genome-wide significant results, at least without strong a priori evidence. However, at the time of writing, data on COVID-19 host genetics were extremely scarce, and given the global health emergency created by the pandemic, and the importance of dementia as a risk factor for severe COVID-19, we believe our work was a useful contribution, with early publication inviting others to examine this potential risk mechanism.

The second issue raised by Nikogosov et al. relates to the UK Biobank providing data on whether the patients tested for COVID-19 were hospital inpatients, and suggesting that the valid comparison should have been between inpatient positives and positives not admitted to hospital. However, the data available at the time were actually derived from the test samples sent to laboratories, stating only where the patient was when the test sample was taken (4). Thus, patients may have been recorded as being in a non-inpatient setting when tested but could have been admitted to hospital soon after. Therefore, the suggested design from Nikogosov et al. would not have been measuring what they suggest, likely biasing the association to the null (5). We note that the only published GWAS to date compared cases with respiratory failure to convenient controls regardless of COVID-19 test results, including blood donors randomly selected for the study and genotyped controls from previous studies (6). Our comparison with the rest of the UK Biobank participants (negative/unknown), therefore, provided a less-selected control group without evidence of COVID-19 and had better statistical power compared to the outcome of inpatient positives versus non-inpatient positives.

## Conflict of Interest

C.L.K. and D.M. are supported by an R21 grant (R21AG060018) funded by National Institute on Aging, National Institute of Health, USA. D.M. also is supported by the University of Connecticut School of Medicine. 

## References

[1] Kuo C-L, Pilling LC, Atkins JL, et al ApoE e4e4 genotype and mortality with COVID-19 in UK Biobank [published online ahead of print July 4, 2020]. J Gerontol A Biol Sci Med Sci. 2020. doi:10.1093/gerona/glaa169 PMC7337688 32623451

[2] Kuo C-L, Pilling LC, Atkins JL, et al APOE e4 genotype predicts severe COVID-19 in the UK Biobank community cohort [published online ahead of print May 26, 2020]. J Gerontol A Biol Sci Med Sci. 2020. doi:10.1093/gerona/glaa131 PMC7314139 32451547

[3] Atkins JL, Masoli JAH, Delgado J, et al Preexisting comorbidities predicting COVID-19 and mortality in the UK Biobank community cohort [published online ahead of print July 20, 2020]. J Gerontol A Biol Sci Med Sci. 2020. doi:10.1093/gerona/glaa183 PMC7454409 32687551

[4] Armstrong J, Rudkin JK, Allen N, et al Dynamic linkage of COVID-19 test results between Public Health England’s Second Generation Surveillance System and UK Biobank. Microb Genomics. 2020;6(7). doi:10.0.4.75/mgen.0.000397 PMC7478634 32553051

[5] Magder LS, Hughes JP Logistic regression when the outcome is measured with uncertainty. Am J Epidemiol. 1997;146:195–203. doi:10.1093/oxfordjournals.aje.a009251 9230782

[6] Ellinghaus D, Degenhardt F, Bujanda L, et al. Genomewide association study of severe COVID-19 with respiratory failure [published online ahead of print June 17, 2020]. N Engl J Med. 2020. doi:10.1056/NEJMoa2020283 PMC7315890 32558485

